# The influence of the -94 *Ins/Del* ATTG polymorphism of NFkB on the anti-CCP antibody levels in patients with rheumatoid arthritis

**DOI:** 10.1097/MD.0000000000028301

**Published:** 2021-12-17

**Authors:** Miriam Fabiola Ayón-Pérez, Jonathan Joseph Topete-Córdoba, Juan Manuel Agraz-Cibrián, Liliana Ortiz-Martínez, Ma. de Jesús Durán-Avelar, Alejandro Vázquez-Reyes, Norberto Vibanco-Pérez, Jorge Gutiérrez-Franco, José Francisco Zambrano-Zaragoza

**Affiliations:** a Unidad Academica de Ciencias Quimico Biológicas y Farmaceuticas-Universidad Autonoma de Nayarit, Tepic, Nayarit, Mexico; b Maestria en Salud Pública, Universidad Autonoma de Nayarit, Tepic, Nayarit, Mexico; c Clinica de Reumatologia, Servicio de Medicina Interna, Instituto Mexicano del Seguro Social HGZ No. 1, Tepic, Nayarit, Mexico.

**Keywords:** anti-CCP, NF-kB, polymorphism, rheumatoid arthritis

## Abstract

Rheumatoid arthritis (RA) is an autoimmune disease characterized by an inflammatory process that affects mainly synovial tissue in joints, and by the production of cyclic citrullinated peptides (anti-CCP) antibodies. In the inflammatory process the regulation of the nuclear factor kappa B (NFkB) transcription factor activation is a key point in the production of inflammatory cytokines. On the other hand, polymorphisms in several genes could contribute to the promotion of the inflammatory process observed in RA, and the association of the rs28362491 polymorphism in the NFkB gene with RA has been studied in different population. Therefore, it could be one of the interest targets to analyze their association with RA in a Mexican population.

This is a case-control study to determine the influence of rs28362491 in the NFkB gene on RA and on clinical features of this disease, such as anti-CCP antibody levels, Disease Activity Score, and Health Assessment Questionnaire-Disability Index.

The genotype of rs28362491 in the NFkB gene was determined in 140 RA patients and 135 healthy controls using the polymerase chain reaction-restriction fragment length polymorphism method with the enzyme *PflMI*. The following clinical variables were also determined: anti-CCP levels, Disease Activity Score, and Spanish version of the Health Assessment Questionnaire Disability-Index.

Although no association of the polymorphism as a risk/protection factor with RA was found, the RA patients who carried the Ins/Ins genotype showed higher anti-CCP levels, while those with the Del/Del genotype showed higher Spanish version of the Health Assessment Questionnaire-Disability Index levels, compared to the other genotypes.

The NFkB -94 Ins/Del ATTG (rs28362491) polymorphism is, therefore, associated with higher levels of anti-CCP antibodies, though no significant association as a risk or protection factor in RA cases was identified.

## 1 Introduction

Rheumatoid arthritis (RA) is an autoimmune disease characterized by chronic inflammation in the synovial tissue of joints.^[1]^ RA affects approximately 1% of the world's population, but prevalence in Mexico is around 1.6%.^[2]^ The activity of this disease can be evaluated using the Disease Activity Score (DAS28)^[3]^ and the severity by the Health Assessment Questionnaire-Disability Index (HAQ-DI).^[4]^

Although the etiology of RA remains unknown, genetic and environmental factors have been shown to be involved in disease development.^[5]^ Therefore, these factors could be implicated, as well, in the loss of immunological tolerance that leads to the production of anti-cyclic citrullinated peptides (anti-CCP) and rheumatoid factor antibodies,^[5]^ two of the main serological characteristics of RA.

Various studies have successfully identified alleles that govern RA susceptibility, especially genetic variants in the major histocompatibility complex,^[6]^ though a contribution of other genetic polymorphisms to signal transducer factors, cytokines, and other receptor genes has also been associated with RA.^[7]^

Signal transduction pathways provide an intracellular mechanism through which cells respond and adapt to environmental stress.^[8]^ One of the intracellular signaling pathways that shows high activity in RA synovial joints is the nuclear factor kappa B (NFkB), which is involved in regulating proinflammatory cytokine production.^[9]^ The NFkB family plays a central role in inflammation because its members can induce the expression of various proinflammatory cytokines.^[10,11]^

The nuclear factor NFkB pathway has been considered a proinflammatory signaling pathway because of its involvement in the expression of proinflammatory genes, including cytokines, chemokines, adhesion molecules, cyclooxygenase (Cox), and inducible nitric oxide synthase (iNOS).^[12]^

The NFkB1 gene is located on the 4q21 chromosome. The -94 Ins/Del ATTG (rs28362491) polymorphism is situated in the promotor region of the NFkB1 gene and is a functional polymorphism. Studies have described that the Ins allele induces a higher expression of NFkB1 compared to the Del allele.^[13]^ NFkB is also overexpressed in the synovia of RA patients.^[12]^

The uncontrolled activation of the NFkB signaling pathway has been involved in promotion of the inflammatory response in different autoimmune disease.^[14–17]^ The association of the rs28362491 in the NFkB gene with RA has been reported by different groups with contradictory results,^[18–20]^ in where the minor allele appears to be dependent of the studied population, and the association of this polymorphism on the clinical features, such as DAS28 and HAQ-DI, have showed also different results.

Describing new susceptibility biomarkers for RA in different populations is important because of their potential usefulness for early diagnosis and in evaluating treatment response in RA patients. In this context, NFkB may be a good candidate due to its involvement in the pathogenesis of RA. The aim of this study, then, was to determine the influence of rs28362491 on RA and on the clinical features of this disease, such as anti-CCP antibody levels, DAS28, and HAQ-DI.

## 2 Materials and methods

### 2.1 Patients and controls

A total of 140 consecutive blood samples from RA patients diagnosed according to the American College of Rheumatology/European League against Rheumatism 2010 criteria in the Rheumatology Clinic of the Internal Medicine Service at the *Instituto Mexicano del Seguro Social* were included in the analysis. DAS28-C reactive protein and HAQ-DI were determined by a qualified rheumatologist. The control group consisted of 135 unrelated healthy subjects, controlling similar proportion of age and gender to those of case group. Sample size was determined using the EpiDAt v4.1 developed by the Consellería de Sanidade de la Xunta de Galicia with the support of PAHO-WHO, considering an odds ratio (OR) = 2.0, 95% confidence, and 80% power.

All subjects were residents of Nayarit, Mexico, 18 years old or older, with no other rheumatic or inflammatory diseases, and gave their written consent in accordance with the Helsinki Declaration.^[21]^ The study was approved by the local ethics committee from Instituto Mexicano del Seguro Social, Tepic Nayarit Mexico (protocol number: 1802 approved on March 25, 2013).

### 2.2 Anti-CCP antibody levels

Anti-CCP antibody levels were determined by enzyme-linked immunosorbent assay, as outlined in Duran-Avelar et al.^[22]^

### 2.3 Genotyping of the rs28362491

Genomic DNA was obtained using the Easy-DNA kit (Invitrogen) following the manufacturer's instructions. The genotype of each sample was obtained by polymerase chain reaction (PCR)-restriction fragment length polymorphism, according to the method originally described by Senol Tuncay et al^[12]^ with a few modifications. Every reaction was performed with 0.5 μM of each primer –forward: 5′-TGG GCA CAA GTC GTT TAT GA-3′ and reverse: 5′-CTG GAG CCG GTA GGG AAG-3′– 0.5 mM of dNTPs, 2 mM of MgCl2, 2.5 μL of PCR 10× buffer, 2 U of Taq polymerase (Invitrogen), and 200 ng of DNA, in a final volume of 25 μL. The thermocycler program was set at 30 seconds at 98°C, 40 cycles of 5 seconds at 98°C, 5 seconds at 65°C, and 5 seconds at 72°C, followed by a final extension of 5 minutes at 72°C. The expected size of the PCR product was 285/280 bp for the Ins/Del alleles.

The polymorphism was detected using 5 μL of the PCR product, digested with 3 U of the *PfIMI* (Takara) enzyme and left overnight at 37°C. The digestion products were observed in a 2% agarose gel. The Ins allele (exscinded by *PfIMI*) appeared at 45 and 245 bp, the Del allele at 280 bp.

### 2.4 Statistical analyses

Risk estimation was calculated by determining the OR and the 95% confidence interval of the OR (95%CI) using the WinEpi tool (http://www.winepi.net). The Hardy-Weinberg equilibrium was determined by a chi-square test. The comparison of anti-CCP, DAS28, and HAQ-DI levels in RA patients grouped according to their rs28362491 genotype was done with the Kruskall-Wallis test with 95% confidence.

## 3 Results

Table 1 shows the characteristics of the subjects included in the study. Most of the RA patients and controls were female (91.4 and 95.4%, respectively).

**Table 1:** Clinical features of patients and controls.

	RA patients	Controls
N (F/M)	140 (128/12)	135 (129/6)
Age (M ± SD)	49.4 ± 11.31	43 ± 13.8
Anti-CCP-n (%)	37 (26.4)	^∗^
Anti-CCP+n (%)	103 (73.6)	^∗^
Anti-CCP^†^ (U/mL)=	126.04 ± 584.08	^∗^
DAS28^†^	4.2 ± 1.8	^∗^
sHAQ-DI^†^	1.0 ± 0.6	^∗^

Anti-CCP = anti-cyclic citrullinated peptides, DAS28 = Disease Activity Scores using 28-joint counts, RA = rheumatoid arthritis, sHAQ-DI = Spanish version of the Health Assessment Questionnaire Disability Index.

∗ Not done.

† Mean ± SD.

After genotyping, risk estimation was determined in distinct genetic models. As Table 2 shows, no significant association of the rs28362492 with RA was found in the genetic models analyzed.

**Table 2:** Association of the -94 Ins/Del ATTG (rs28362492) with rheumatoid arthritis.

		Frequency			
Genetic model	Genotype	RA patients	Controls	OR (CI95%)	*P*
Co	Del/Del	44	43	ref	
	Ins/Del	84	73	0.6172 (0.2675–1.4241)	.2560
	Ins/Ins	12	19	1.1245 (0.6656–1.8999)	.6608
Do	Del/Del	44	43	1.0198 (0.6128–1.6970)	.9399
	Ins/Del+Ins/Ins	96	92		
Re	Ins/Ins	12	19	0.5724 (0.2663–1.2302)	.1492
	Ins/Del+Del/Del	128	116		
Additive	Ins	108	111	1.1118 (0.7901–1.5645)	.5430
	Del	172	159		

CI95% = confidence interval, Co = codominant, Do = dominant, OR = odds ratio, RA = rheumatoid arthritis, Re = recessive.

**Figure 1:**
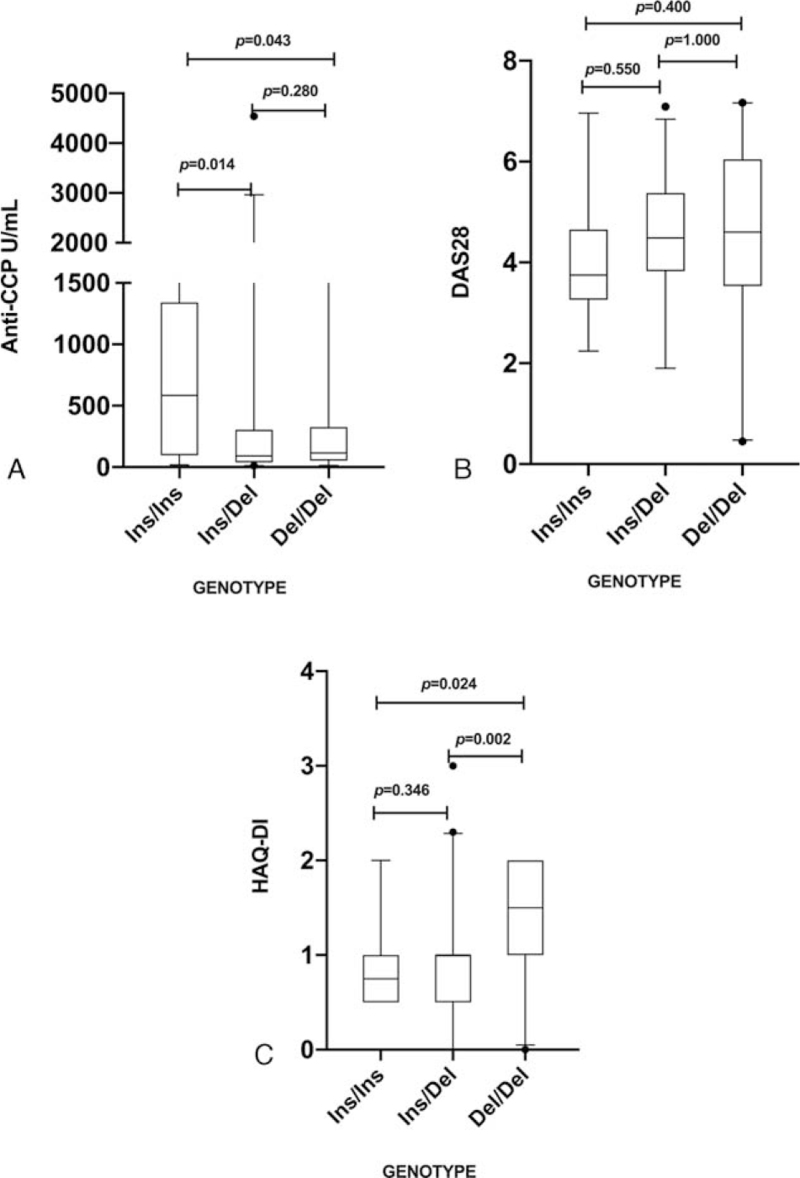
Influence of the rs28362491 polymorphism on (A) anti-CCP antibody levels; (B) DAS28; and (C) sHAQ-DI, in RA patients grouped according to their genotype. Comparison performed with the Mann-Whitney *U* test. Anti-CCP = anti-cyclic citrullinated peptides, DAS28 = Disease Activity Score, RA = rheumatoid arthritis, sHAQ-DI = Spanish version of the Health Assessment Questionnaire Disability Index.

Considering that the rs28362492 is a functional polymorphism, we tested whether some of its genotypes could be associated with lower or higher levels of anti-CCP, DAS28, or HAQ-DI. The RA patients with the Ins/Ins genotype showed higher anti-CCP levels than those with the Ins/Del and Del/Del genotypes (*P* = .043 and .014, respectively [Fig. 1A]). RA patients with the Del/Del genotype, meanwhile, showed significantly higher HAQ-DI values than those of the patients with the Ins/Ins or Ins/Del genotypes (*P* = .0245 and .0028, respectively, Fig. 1C). No association of the different genotypes with DAS28 was found (Fig. 1B).

## 4 Discussion

RA is a multifactorial autoimmune disease characterized by a chronic inflammatory process^[7]^ in which cytokines such as TNFα, IL-1, and IL-6 are involved and induce activation of the NFkB transcription factor.^[11,23]^ The NFkB -94 Ins/Del ATTG polymorphism (rs28362491) has been associated with various inflammatory diseases, including cancer^[24]^ and several autoimmune diseases.^[25]^

In this study of a Mexican population, we did not find an association of rs28362491 with RA. These results agree with those of Bogunia-Kubik et al,^[18]^ Gębura et al,^[26]^ and Gomes da Silva et al,^[27]^ who also failed to determine an association of rs28362491 with RA in Polish and Brazilian populations, respectively, though Elkhawaga et al^[20]^ reported that the Del/Del genotype was associated as a risk factor for RA in an Egyptian population. This polymorphism, in particular the Ins allele, has also been associated with other inflammatory diseases, such as Behçet disease^[28]^ and psoriasis,^[29]^ while the Ins/Ins genotype has been associated with a higher risk of developing colorectal cancer.^[30]^

Our next step consisted in analyzing the levels of anti-CCP antibodies in RA patients grouped according to their rs28362491 genotypes. As Figure 1 shows, patients with the Ins/Ins genotype had higher anti-CCP levels than those with the Del/Del (*P* = .043) or Ins/Ins genotypes (*P* = .014). Studies have reported that the Ins allele increases expression of NFkB^[13]^ so, given that NFkB regulates numerous pro-inflammatory cytokines (including TNF-a, IL-1b, IL-6, or IL-7 involved in the activation and recruitment of inflammatory cells^[18]^), this inflammatory environment could lead to non-specific citrullination^[31]^ that promotes the production of anti-CCP antibodies in RA patients. Moreover, NFkB has been also involved in dendritic, B and T cell activation,^[17]^ and B cells play an important role in RA, because of the production of anti-CCP antibodies.^[32]^ Therefore, the association of the rs28362491 polymorphism in the NFkB gene with anti-CCP antibody levels observed could be explained through the influence of the NFkB on IL-6 production, non-specific citrullination and B cell activation. However, it has been also reported not association of this polymorphism with anti-CCP in Egyptian population,^[19]^ suggesting that heterogeneity in different populations could affect the influence of this polymorphism on anti-CCP in RA.

Upon comparing the HAQ-DI values in RA patients grouped according to their rs28362491 genotype, those that carried the Del/Del genotype showed the highest levels, compared to those with the Ins/Del and Ins/Ins genotypes (*P* = .0028 and .0245, respectively). In concordance with our results, Elkhawaga et al^[20]^ reported that this polymorphism was associated with disease severity and progression in RA patients in Egypt. However, no association of this polymorphism with DAS28 or HAQ was determined in a Brazilian population.^[27]^

Regarding the association of this polymorphism with DAS28 scores, we did not find any association, according to Gomes da Silva et al,^[27]^ who reported no association of this polymorphism with DAS28 in a Brazilian population. Contrasting our results, Elkhawaga et al^[20]^ reported association of this polymorphism with DAS28 and disease progression, however, they included RA patients not receiving synthetic or biological disease-modifying anti-rheumatic drugs.

Some limitation in the study such as the onset and treatment, that could affect the progression and severity of the disease, that were not controlled variable in this study.

This study suggest that the -94 ATTG polymorphism could be associated with RA thought the production of anti-CCP antibodies, however, it is important to consider the heterogeneity of the population and explore this association in a prospective study to confirm this conclusion.

## 5 Conclusions

In conclusion, the NFkB -94 Ins/Del ATTG (rs28362491) polymorphism is associated with higher levels of anti-CCP antibodies, though no association with RA as a risk or protection factor was found.

## Acknowledgments

Authors thanks to Indra Karym Juárez Sánchez for their technical assistance

## Author contributions

**Conceptualization:** Jose Francisco Zambrano-Zaragoza.

**Formal analysis:** Miriam Fabiola Ayón-Pérez, Juan Manuel Agraz-Cibrián, Ma. de Jesús Durán-Avelar, Alejandro Vázquez-Reyes, Norberto Vibanco-Pérez, Jorge Gutiérrez-Franco, Jose Francisco Zambrano-Zaragoza.

**Funding acquisition:** Jose Francisco Zambrano-Zaragoza.

**Investigation:** Jonathan Joseph Topete-Córdoba, Liliana Ortiz-Martínez.

**Methodology:** Juan Manuel Agraz-Cibrián, Jose Francisco Zambrano-Zaragoza.

**Project administration:** Jose Francisco Zambrano-Zaragoza.

**Supervision:** Juan Manuel Agraz-Cibrián, Alejandro Vázquez-Reyes, Jose Francisco Zambrano-Zaragoza.

**Validation:** Juan Manuel Agraz-Cibrián, Jorge Gutiérrez-Franco, Jose Francisco Zambrano-Zaragoza.

**Writing – original draft:** Miriam Fabiola Ayón-Pérez, Jonathan Joseph Topete-Córdoba, Juan Manuel Agraz-Cibrián, Liliana Ortiz-Martínez, Ma. de Jesús Durán-Avelar, Alejandro Vázquez-Reyes, Norberto Vibanco-Pérez, Jorge Gutiérrez-Franco, Jose Francisco Zambrano-Zaragoza.

**Writing – review & editing:** Miriam Fabiola Ayón-Pérez, Jonathan Joseph Topete-Córdoba, Juan Manuel Agraz-Cibrián, Liliana Ortiz-Martínez, Ma. de Jesús Durán-Avelar, Alejandro Vázquez-Reyes, Norberto Vibanco-Pérez, Jorge Gutiérrez-Franco, Jose Francisco Zambrano-Zaragoza.

## References

[1] Calabresi E Petrelli F Bonifacio AF Puxeddu I Alunno A. One year in review 2018: pathogenesis of rheumatoid arthritis. Clin Exp Rheumatol 2018;36:175–84. 29716677

[2] Burgos-Vargas R Catoggio LJ Galarza-Maldonado C Ostojich K Cardiel MH. Current therapies in rheumatoid arthritis: a Latin American perspective. Reumatol Clin 2013;9:106–12. 23337169 10.1016/j.reuma.2012.09.001

[3] van Riel PL Renskers L. The Disease Activity Score (DAS) and the Disease Activity Score using 28 joint counts (DAS28) in the management of rheumatoid arthritis. Clin Exp Rheumatol 2016;34: (5 Suppl 101): S40–4. 27762189

[4] Cardiel MH Abello-Banfi M Ruiz-Mercado R Alarcon-Segovia D. How to measure health status in rheumatoid arthritis in non-English speaking patients: validation of a Spanish version of the Health Assessment Questionnaire Disability Index (Spanish HAQ-DI). Clin Exp Rheumatol 1993;11:117–21. 8508553

[5] Sanchez-Ramon S Lopez-Longo F Carreno L. Interleukins network in rheumatoid arthritis pathophysiology: beyond proinflammatory cytokines. Reumatologia clinica 2011;6:S20–4. 10.1016/j.reuma.2010.11.010 21794767

[6] Giannini D Antonucci M Petrelli F Bilia S Alunno A Puxeddu I. One year in review 2020: pathogenesis of rheumatoid arthritis. Clin Exp Rheumatol 2020;38:387–97. 32324123

[7] Perricone C Ceccarelli F Valesini G. An overview on the genetic of rheumatoid arthritis: a never-ending story. Autoimmun Rev 2011;10:599–608. 21545847 10.1016/j.autrev.2011.04.021

[8] Sweeney SE Firestein GS. Signal transduction in rheumatoid arthritis. Curr Opin Rheumatol 2004;16:231–7. 15103250 10.1097/00002281-200405000-00011

[9] Alam J Jantan I Bukhari SNA. Rheumatoid arthritis: recent advances on its etiology, role of cytokines and pharmacotherapy. Biomed Pharmacother 2017;92:615–33. 28582758 10.1016/j.biopha.2017.05.055

[10] Baldwin AS Jr. The NF-κB and IκB proteins: new discoveries and insights. Annu Rev Immunol 1996;14:649–81. 8717528 10.1146/annurev.immunol.14.1.649

[11] Schett G. Erosive arthritis. Arthritis Res Ther 2007;9:S2. 10.1186/ar2166 PMC1924517 17634141

[12] Senol Tuncay S Okyay P Bardakci F. Identification of NF-κB1 and NF-κBIΑ polymorphisms using PCR–RFLP assay in a Turkish population. Biochem Genet 2010;48:104–12. 19941056 10.1007/s10528-009-9302-y

[13] Eskandari-Nasab E Hashemi M Ebrahimi M Amininia S. The functional 4-bp insertion/deletion ATTG polymorphism in the promoter region of NF-KB1 reduces the risk of BC. Cancer Biomark 2016;16:109–15. 26835711 10.3233/CBM-150546 PMC13016534

[14] Cen H Zhou M Leng RX. Genetic interaction between genes involved in NF-κB signaling pathway in systemic lupus erythematosus. Mol Immunol 2013;56:643–8. 23911423 10.1016/j.molimm.2013.07.006

[15] Chatzikyriakidou A Kyriakou A Meltzanidou P Lambropoulos A Patsatsi A. Association of NFKB1-94ATTG ins/del polymorphism (rs28362491) with pemphigus vulgaris. Exp Dermatol 2019;28:972–5. 31077459 10.1111/exd.13957

[16] Yenmis G Oner T Cam C. Association of NFKB1 and NFKBIA polymorphisms in relation to susceptibility of Behçet's disease. Scand J Immunol 2015;81:81–6. 25367031 10.1111/sji.12251

[17] Zahednasab H Mesbah-Namin SA Sahraian MA Balood M Doosti R. Relationship between NF-κB1-94 ins/del ATTG polymorphism and susceptibility of multiple sclerosis in Iranian MS patients. Neurosci Lett 2013;545:46–9. 23618653 10.1016/j.neulet.2013.04.014

[18] Bogunia-Kubik K Wysoczańska B Piątek D Iwaszko M Ciechomska M Świerkot J. Significance of polymorphism and expression of miR-146a and NFkB1 genetic variants in patients with rheumatoid arthritis. Arch Immunol Ther Exp (Warsz) 2016;64:131–6. 28083614 10.1007/s00005-016-0443-5 PMC5334424

[19] Gębura K Świerkot J Wysoczańska B. Polymorphisms within genes involved in regulation of the NF-κB pathway in patients with rheumatoid arthritis. Int J Mol Sci 2017;18:1432. 28677621 10.3390/ijms18071432 PMC5535923

[20] Elkhawaga SY Gomaa MH Elsayed MM Ebeed AA. NFKB1 promoter -94 insertion/deletion ATTG polymorphism (rs28362491) is associated with severity and disease progression of rheumatoid arthritis through interleukin-6 levels modulation in Egyptian patients. Clin Rheumatol 2021;40:2927–37. 33459954 10.1007/s10067-021-05584-z

[21] Association GAotWM. World Medical Association Declaration of Helsinki: ethical principles for medical research involving human subjects. JAMA 2014;81:14. 25951678

[22] Duran-Avelar MJ Vibanco-Perez N Hernandez-Pacheco RR Castro-Zambrano AD Ortiz-Martinez L Zambrano-Zaragoza JF. STAT4 rs7574865 G/T polymorphism is associated with rheumatoid arthritis and disease activity, but not with anti-CCP antibody levels in a Mexican population. Clin Rheumatol 2016;35:2909–14. 27234231 10.1007/s10067-016-3320-z

[23] Riancho J Delgado-Calle J. Osteoblast-osteoclast interaction mechanisms. Reumatol Clin 2011;7:S1–4. 10.1016/j.reuma.2011.03.003 21924211

[24] Cartwright T Perkins ND Wilson CL. NFKB1: a suppressor of inflammation, ageing and cancer. FEBS J 2016;283:1812–22. 26663363 10.1111/febs.13627

[25] Karban AS Okazaki T Panhuysen CI. Functional annotation of a novel NFKB1 promoter polymorphism that increases risk for ulcerative colitis. Hum Mol Genet 2004;13:35–45. 14613970 10.1093/hmg/ddh008

[26] Gębura K Świerkot J Wysoczańska B. Polymorphisms within genes involved in regulation of the NF-(B pathway in patients with rheumatoid arthritis. Int J Mol Sci 2017;18:1432. 28677621 10.3390/ijms18071432 PMC5535923

[27] Gomes da Silva IIF Lima CAD Monteiro MLA. IL1β, IL18, NFKB1 and IFNG gene interactions are associated with severity of rheumatoid arthritis: a pilot study. Autoimmunity 2020;53:95–101. 31992083 10.1080/08916934.2019.1710831

[28] Yenmis G Oner T Cam C. Association of NFKB 1 and NFKBIA polymorphisms in relation to susceptibility of Behçet's disease. Scand J Immunol 2015;81:81–6. 25367031 10.1111/sji.12251

[29] Li H Gao L Shen Z. Association study of NFKB1 and SUMO4 polymorphisms in Chinese patients with psoriasis vulgaris. Arch Dermatol Res 2008;300:425. 18330586 10.1007/s00403-008-0843-4

[30] Suzairi MSM Tan SC Aizat AAA. The functional -94 insertion/deletion ATTG polymorphism in the promoter region of NFKB1 gene increases the risk of sporadic colorectal cancer. Cancer Epidemiol 2013;37:634–8. 23806437 10.1016/j.canep.2013.05.007

[31] Fert-Bober J Darrah E Andrade F. Insights into the study and origin of the citrullinome in rheumatoid arthritis. Immunol Rev 2020;294:133–47. 31876028 10.1111/imr.12834 PMC8061312

[32] Mateen S Zafar A Moin S Khan AQ Zubair S. Understanding the role of cytokines in the pathogenesis of rheumatoid arthritis. Clin Chim Acta 2016;455:161–71. 26883280 10.1016/j.cca.2016.02.010

